# Predicting influenza with dynamical methods

**DOI:** 10.1186/s12911-016-0371-7

**Published:** 2016-10-19

**Authors:** Linda Moniz, Anna L. Buczak, Ben Baugher, Erhan Guven, Jean-Paul Chretien

**Affiliations:** 1 Johns Hopkins University Applied Physics Laboratory, 11100 Johns Hopkins Road, Laurel, MD USA; 2 Armed Forces Health Surveillance Branch, Defense Health Agency, Silver Spring, MD USA

**Keywords:** Influenza, Prediction, Analogues

## Abstract

**Background:**

Prediction of influenza weeks in advance can be a useful tool in the management of cases and in the early recognition of pandemic influenza seasons.

**Methods:**

This study explores the prediction of influenza-like-illness incidence using both epidemiological and climate data. It uses Lorenz’s well-known Method of Analogues, but with two novel improvements. Firstly, it determines internal parameters using the implicit near-neighbor distances in the data, and secondly, it employs climate data (mean dew point) to screen analogue near-neighbors and capture the hidden dynamics of disease spread.

**Results:**

These improvements result in the ability to forecast, four weeks in advance, the total number of cases and the incidence at the peak with increased accuracy. In most locations the total number of cases per year and the incidence at the peak are forecast with less than 15 % root-mean-square (RMS) Error, and in some locations with less than 10 % RMS Error.

**Conclusions:**

The use of additional variables that contribute to the dynamics of influenza spread can greatly improve prediction accuracy.

## Background

### Introduction

Currently and historically, seasonal influenza epidemics caused by influenza A and influenza B viruses occur worldwide in the winter months in temperate climates. In some individuals, they cause severe illness; 250,000-500,000 deaths are estimated to occur from influenza or its complications each year [1]. In addition to seasonal influenza, novel infections occur occasionally. Because these novel influenza strains may not be affected by existing antibodies in individuals, they can cause pandemic outbreaks.

Countermeasures such as development of additional vaccines and hospital resource management can be greatly aided by accurate forecasts of the number of cases and the peak of the influenza season. In addition, accurate forecasts can give warning of the emergence of a pandemic or the presence of a strain for which there is little immunity from the year’s influenza vaccine.

Although seasonal influenza is predictably periodic, influenza spread is influenced by many factors, including the strain(s), the match of the seasonal vaccine to the strains, the immunization rate, the weather [2] and the contact of individuals with others. Many of these data are not easy, if even possible, to obtain, and the exact relationships between the data and influenza incidence are not known and are likely to be evolving [2]. Thus we turn to a data-driven model for prediction in order to reduce complexity and make the model reflect local variation in the factors affecting influenza transmission.

### Related work

A survey of influenza forecasting methods [3] yielded 35 publications organized into categories based on the epidemiological application – population-based, medical facility-based, and forecasting regionally or globally. Within these categories, the forecasting methods varied along with the types of data used to make the forecast. Roughly half of the publications used statistical approaches without explicit mechanistic models and the other half used epidemiological models. Three of these models used meteorological predictors.

In this study, we model directly from the data (time series consisting of weekly incidence geographically aligned with multiple facilities) and use meteorological data to enrich the model. None of the models surveyed in [3] used both the Method of Analogues and meteorological data to forecast influenza in a population.

Typically data on the current number of influenza cases reported by the Centers for Disease Control ([4]; one of the more accurate geographically tagged data sets) has a one-week lag. In order to predict 4 weeks ahead of the current date, one uses data up to one week before the current date. This translates, in reality, to a 5-week prediction horizon for a prediction 4 weeks in the future. For the remainder of the paper we will refer to this as a 4-week prediction. Similarly, most climate data for the current date is not available in a format for which acquisition can be automated immediately; for most there is a lag of about one week. Our goal is to predict influenza incidence (number of influenza cases/total number of health-care visits) 4 weeks ahead of the current date, using only data available up to the current time, that is, using both incidence and climate data from the week before.

This study was part of a team effort to predict the height of the peak, the timing of the peak and the total cases in an influenza season. This paper addresses the height of the peak and the total cases in a season. Another paper (see [5]) uses machine-learning methods to predict the timing of the peak.

## Methods

### Overview

#### The method of analogues

The method of analogues is a prediction method originally proposed by Lorenz [6] to predict weather patterns, but more recently used by Viboud et al. [7] for influenza prediction. The idea is the following: Weather (or the spread of influenza) is assumed to be a continuous, but not necessarily linear, deterministic process. *Deterministic* dictates that a future discrete-time observation depends only on the observation immediately prior to that observation and an underlying functional process. *Continuous* dictates that “nearby points map to nearby points,” meaning that although the process may be nonlinear and therefore not predictable over long periods of time, previous sequences of historical observations that are close to current sequences of observations should yield “close” subsequent observations. The principles both of determinism and of continuity allow us to use sequences of observations to predict other sequences of observations. This is the reasoning behind the method of analogues.

**Fig. 1:**
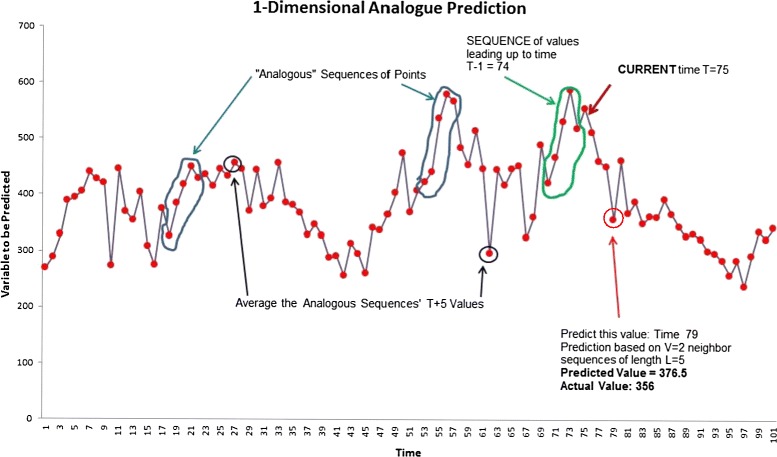
The blue-circled sequences of points are “analogous” to the green sequence for which a prediction is desired. Average the black-arrow designated values to arrive at the prediction

The method of analogues is illustrated in Fig. 1. A prediction is desired for the point with the red arrow. Sequences of points are found which are close (Euclidean-distance) to the green-circled sequence prior to the desired prediction point. The time-advanced values are located in the time series. The values for the black-circled points are averaged to arrive at the prediction for the point.

The method of analogues begins with the time of the point to be predicted, call it *x*. For *h*- week predictions, the sequence of values at time points leading to *h* weeks **before** *x* is recorded. Call this sequence ***T***. Sequences ***S*** of points closest (in distance) to ***T*** are found in the historical data, but recent data are not included in the search. The points that occur *h* weeks after the historical sequences are used to predict *x*. These points are averaged (in this implementation with equal weights, but there are many options for weights based on distance from the sequence ***T*** or time-distance from ***T***).

Several factors dictate the accuracy of the method of analogues. Obviously, the longer the historic time series and the faster the data rate, the better the characterization of the deterministic process that is producing the observations. The parameters *l* and *v*, the length of the prediction sequence ***S*** and the number of sequences can also greatly influence the quality of the prediction for any prediction horizon *h*. Shorter prediction horizons *h*, as with any prediction method, typically produce more accurate forecasts.

### Novel applications of the method of analogues

Previous implementations of the method of analogues to influenza forecasting ([7, 8]) treated the time series as a one-dimensional process, that is, any analogues are determined as sequences of closest (based on the metric used) sequences of points to the sequence *T*. In this implementation, we recognize that although the measurement of incidence is a one-dimensional time series, it is really a projection of a *multidimensional* continuous, deterministic process for which many of the variables are not available. Theoretically, inclusions of additional variables that are known to directly affect the transmission or viability of the influenza virus are then relevant to the dynamics of influenza incidence. This is the basis for our study.

One option for analysis of dynamics that is used extensively in physical experiments is phase-space reconstruction. An evolving process is measured for a length of time and then the measurements are used to reconstruct the entire state-space. Theorems guarantee the faithful reproduction of the state-space directly from one variable using delay-coordinate embedding [9].

Typically the reconstruction of dynamics from time series via delay-coordinate embedding requires a long time series to populate the state-space. One rule of thumb is about 10,000 points per dimension. Unfortunately, a typical epidemiological time series of weekly data is much shorter. Reconstruction of a faithful representation of influenza transmission dynamics from influenza incidence alone is not practical or possible. The short time series severely restrict what can be reconstructed from the time series or its delay coordinates. However, the theory behind state-space reconstruction does yield another avenue. If an additional variable is known to affect the dynamics of the variable in question, its measurements, when sufficiently orthogonal to the existing variable, can be used to further describe the dynamics of the influenza transmission process. The degree to which the variable is orthogonal can be tested using either mutual information or the continuity test (see e.g. [10, 11]).

A recent study [2] linked influenza transmission to the temperature and relative humidity of the ambient air. Dew point is a weather variable that incorporates both temperature and humidity, and thus should add an additional relevant criterion, independent of influenza incidence, that will aid in selection of analogues that are not only close in the incidence dimension, but also close in the climate dimension. Using another relevant variable describes the *dynamics* of transmission more accurately. These two-dimensional analogous sequences may not be the same as those chosen using only the influenza incidence. However, the *evolution* of these sequences may be closer to the *evolution* of the test sequence ***T*** because the climate variable is included. The prediction for influenza incidence remains the average of only the influenza incidence values.

The time series of points that is used for prediction itself affects the quality of the prediction. If the time series is nearly periodic and has many sequences of points that are close, analogous sequences are close in distance to the test sequence ***T*** and the time-advances of these sequences should be closer. If, however, the time series is noisy or not as obviously periodic, sequences are far-apart, it is more difficult to find multiple analogous sequences and the averages of their time-advances will be farther apart. Thus, it is advantageous to calculate the “closeness” of typical sequences to see how many analogue sequences *v* are supported by the data. We calculate this space scale, which can vary for each time series. This must be carried out on a subset of the data so that predictions are not contaminated by essentially using the same set of data for determination of parameters and for validation of the method. This is comparable to the dividing of data used for machine-learning methods into “test” and “validation” subsets. The space scale parameter is calculated based on the “test” subset; the method is validated using the entire data set. However, predictions are only made for the portion of the data in the “validation” subset.

### Data

The case data for this study include all medical encounters for influenza-like illness (ILI) paid for by the Department of Defense (DoD) in the United States and 4 US territories (at US military treatment facilities and non-military facilities) from December 2000 through April 2013. Data were obtained from the Armed Forces Health Surveillance Center’s [12, 13] Defense Medical Surveillance System, the central repository of medical surveillance data for the US Armed Forces (Center, Defense Medical Surveillance System) which provides near-complete capture of medical encounters for military personnel (who typically use DoD-sponsored healthcare) and incomplete coverage of other DoD healthcare beneficiaries (e.g., retirees and family members of military personnel). ILI was defined using ICD-9 diagnostic codes validated previously using laboratory data [14]. Data were de-identified prior to use by the investigators, and provided as an operational public health activity of the AFHSC in accordance with AFHSC policy, as with mathematical modeling projects using similar AFHSC ILI datasets [15].

The data fields included date of encounter, military treatment facility identifier, gender, age, cohort (service member of other beneficiary, such as spouse of service member), and type of encounter (inpatient or outpatient). We calculated the CDC-epidemiological week [4] for each date. We aggregated age, gender, cohort and type for each military treatment facility, and converted the military epidemiological weeks to CDC-epidemiological weeks. The data aggregation yielded a weekly time series of both ILI visits and of total visits for each military treatment facility. We used the data provided in [16] to then aggregate the data by U.S. state. That is, we included the military treatment facilities in each state in that state’s aggregate data.

We calculate ILI incidence as the number of ILI cases divided by the total number of cases. We excluded data from 10 states and all territories because the time series were incomplete, and aggregated data across facilities within each state in the ILI incidence calculation.

We note that military treatment facilities frequently have fluid movement of personnel among adjacent states. That is, a person who becomes ill is likely to visit the treatment facility that has the earliest available appointment, if that facility is within a reasonable radius of the person’s residence or workplace, whether or not that facility is in the state of residence.

We examined the data set in advance of the analysis via the Method of Analogues. The population for this data set includes active military personnel and their dependents. Thus, it includes all age groups, with a somewhat skewed population in the 20–40 year old range. The data exhibited a jump in total visits after 2006, and a smaller jump in ILI visits for most states, reflecting increased access to healthcare encounter data beginning at that time. Because we used ILI incidence (ILI cases/total cases) in the modeling, the result appeared as a reduction in the ILI incidence after 2006.

The climate data used for this study are weekly mean dew point measurements collected by weather stations. The dew point is the temperature below which the water vapor in a volume of humid air at a given constant barometric pressure will condense into liquid water at the same rate at which it evaporates [17]. Because the dew point is never higher than the temperature, the dew point is a measure of both temperature and humidity. The unit is in degrees Celsius. The source for these data is the National Oceanic and Atmospheric Administration National Climatic Data Center (NOAA NCDC) Quality controlled Climatological Data (QCLCD) [18], downloaded daily from selected weather stations, and averaged weekly to coincide with epidemiological weeks used in the ILI data.

A concurrent study [5] using the same data predicted the *timing* of the peak incidence but that method was not applicable to predicting the number of cases at the peak. This method was able to predict the cases at the peak as well as the total cases.

### Experimental design

We calculated ILI incidence for the 700 time points corresponding to CDC epidemiological weeks that covered the time interval of the data. The ILI incidence is calculated for each week *t* using the formula:

$$ ILI\_ Incidence(t) = \frac{ILI\_ Cases(t)}{Total\_ Cases(t)} $$

For the initial baseline experiment, we calculated the parameters *l* and *v* that optimized the five-time-step-ahead (four-week) predictions for all states’ time series. The parameters chosen were *l* = 7 and *v* = 3, using a parameter sweep. This was done using the first 550 points of the time series. Predictions were made for each week *after* week 550, using prediction horizon *h* = 5 (for a four-week prediction), that is to predict a point at time *t,* the sequence ***T*** was identified as ILI incidence at the times ((t-1)-*l*, (t-1) – (*l*-1), …(t-1)). The test sequence ***T*** of values was used to locate *v* nearest-neighbor sequences ***S*** of length *l* in the time series of ILI incidence, excluding the points from *t*-25 forward in time. Thus, all predictions are *prospective* and simulate the prediction of the future using *only* information that would have been available at the time of the prediction.

Each sequence ***S*** consists of ILI incidence at times ((τ-(*l*-1)) … (τ -2), (τ -1), (τ)) for time τ. We then “advance” each sequence ***S*** and obtain ILI incidence at time (τ + *h*). The values *ILI_Incidence* (τ + h) are averaged for the *v* different sequences to obtain the prediction **p**(*t*). Experiments with different weightings for analogues sequences closer in time or sequence-space did not improve the predictions; thus the average was used. The entire time series of 4-week-ahead predictions of values at points *t* = 550 to *t* = 700 is then reported. This encompasses two years and one partial year. The window *t*-25 was chosen as approximately half of a large oscillation of the time series. This assures that analogous dynamics remain in previous years. This allows results to be validated against the ground truth value at the time of the prediction and provides a reasonable estimate of the accuracy of the method if it is to be used for real-time prediction. Results from this “optimized” set of parameters, along with the parameters *l* and *v* associated with each state, appear in Table 1.

**Table 1:** Results from prediction of influenza incidence in each state for the three prediction years 2010–2013 using the method of analogues with adaptable parameters *l* and *v* as well as the same measures for the naïve prediction (average of the date’s incidence for the previous 4 years)

State	Analogue: % RMS Error in Peak Height:	Analogue: % RMS Error in Area under the Incidence Curve	Correlation Coefficient: Incidence to Prediction	Naïve: % RMS Error in Peak Height	Naïve: % RMS Error in Area under the Incidence Curve
Alabama	16.7	17.2	0.74	25.3	16.6
Alaska	12.7	20.0	0.41	38.0	20.3
Arkansas	14.6	17.4	0.72	29.6	14.2
Arizona	7.6	23.2	0.76	20.9	16.3
California	17.8	26.2	0.68	19.8	12.5
Colorado	3.7	16.0	0.80	19.9	11.5
Connecticut	17.2	8.6	0.62	22.3	5.6
Delaware	17.4	48.6	0.58	21.3	5.9
Florida	9.5	16.4	0.72	20.0	3.3
Georgia	8.3	13.3	0.79	25.8	9.6
Hawaii	11.1	9.6	0.64	28.1	27.2
Illinois	13.5	12.3	0.48	50.8	27.8
Kansas	8.2	17.7	0.63	42.7	11.2
Kentucky	12.5	21.5	0.74	26.5	12.5
Louisiana	10.3	11.6	0.68	23.8	9.1
Maryland	18.3	23.2	0.61	15.5	20.9
Massachusetts	7.6	20.7	0.66	27.7	7.1
Mississippi	10.5	19.0	0.71	30.8	4.6
Missouri	13.3	18.3	0.80	26.7	17.9
Montana	9.8	23.8	0.74	13.9	8.2
Nebraska	18.4	12.7	0.59	37.7	12.4
Nevada	4.2	5.8	0.77	20.3	9.4
New Hampshire	26.7	23.2	0.56	20.9	13.7
New Jersey	13.0	37.6	0.56	13.7	12.7
New Mexico	14.4	17.8	0.76	26.2	1.2
New York	28.8	6.4	0.76	25.1	15.7
North Carolina	19.8	50.0	0.78	22.6	11.4
North Dakota	19.0	15.1	0.57	19.7	11.3
Ohio	7.4	23.8	0.76	28.5	4.8
Oklahoma	8.2	22.7	0.53	28.9	11.2
Pennsylvania	12.4	11.7	0.79	23.7	2.8
Rhode Island	31.5	22.3	0.62	31.6	32.0
South Carolina	18.1	21.4	0.71	31.0	17.7
South Dakota	6.7	13.3	0.71	20.2	17.3
Tennessee	11.2	15.3	0.82	20.4	13.2
Texas	19.4	14.6	0.69	26.3	29.7
Utah	20.1	10.4	0.64	24.5	17.8
Virginia	35.4	17.5	0.66	23.6	12.6
Washington	24.1	17.1	0.70	21.6	18.2
Wyoming	13.2	24.9	0.65	18.4	17.8
Average over All States	14.8	19.2	0.68	25.4	13.7

The second experiment includes dew point in the selection of analogue sequences. For each state, we downloaded the mean dew point from the National Weather Service for the location in the state that exhibited the most complete dew point data, typically the largest city in the state. Each sequence *v* was a sequence of two-dimensional variables (*incidence(t)*, *dew point(t)*) and the search for analogue values was done on the two-dimensional space. The prediction was obtained by averaging *incidence* for the chosen analogue sequences.

### Metrics

To compute the percent RMS error in Peak Height, one peak (highest value) per year is identified in both the data and in the prediction **p**(*t*). The two years and one partial year will be treated as three years for which the peaks are identified. Thus, peak height for the data is calculated to obtain (Peak_data_(1), Peak_data_(2), Peak_data_(3)) and the peak height for the prediction is calculated to obtain (Peak_pred_(1), Peak_pred_(2), Peak_pred_(3)). The RMS difference in peak height for all three years of prediction is then calculated, summed, and divided by the sum of peak heights in the data:

1 $$ RMSDiff=\sqrt{{\displaystyle {\sum}_{i=1}^3}{\left( Pea{k}_{data}(i) - Pea{k}_{pred}(i)\right)}^2}. $$

Percent RMS difference for the *peak height* is then calculated:

2 $$ \% RMSDiff = \sqrt{\frac{1}{3}\left[\frac{pea{k}_{data}(i) - pea{k}_{pred}(i)}{pea{k}_{data}(i)}\right]} $$

We computed additional metrics for the area under the curve (total cases). To compute the percent error in area under the incidence curve (total cases), divisions were made in accordance with the oscillations present in the data. These divisions were: week 551 to week 604 (5/2/2010 to 5/1/2011), week 605 to week 657 (5/8/2011 to 4/29/2012) and week 658 to week 700 (5/6/2012 to 2/24/2013). The total ILI incidence count was computed for the test data and the predicted ILI total case count was computed for the prediction sequence per test division. The RMS error was computed for each division, and the RMS error divided by the total cases for the test data to arrive at the error for the incidence curve, that is,

3 $$ RMSDif{f}_{cases\  per\  year} = \sqrt{\frac{1}{3}{\displaystyle \sum_{i=1}^3}{\left( Area\  Under\  true\  Curve(i)- Area\  under\  prediction(i)\right)}^2}. $$

We computed the RMS total percent error:

4 $$ RMSPercentDif{f}_{cases\  per\  year}=100 \times \frac{RMSDif{f}_{cases\  per\  year}}{mean\left( Cases\  per\  year\right)} $$

We also computed a more dynamic measure, the average running cumulative percent error. This metric measures the average error percent as the total cases are computed prospectively:

5 $$ Running\% Error(i)=100 \times \left[\frac{abs\left({\displaystyle {\sum}_{j=1}^i} FluCases(j) - {\displaystyle {\sum}_{j=1}^i} PredictedFluCases(j)\right)}{{\displaystyle {\sum}_{i=1}^j} FluCases(j)}\right] $$

We average this running error to get

6 $$ AverageRunning\% Error= mean\left( Running\% Error\right). $$

This measures, on average how well the prospective predictions for total cases estimate the true values as they are computed.

## Results

**Fig. 2:**
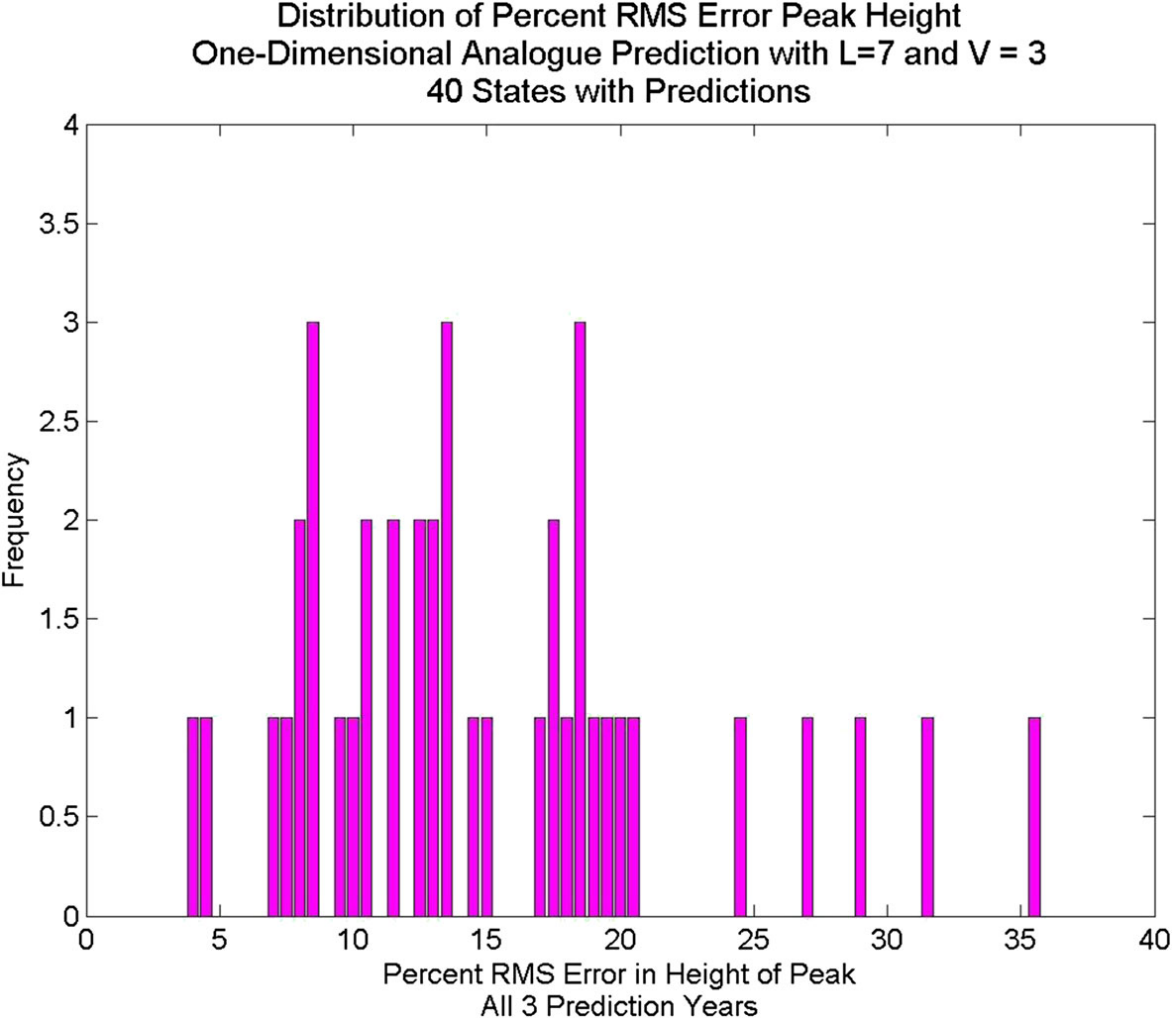
Distribution of percent RMS error in height of yearly peak, with Analogue one-dimensional prediction

**Fig. 3:**
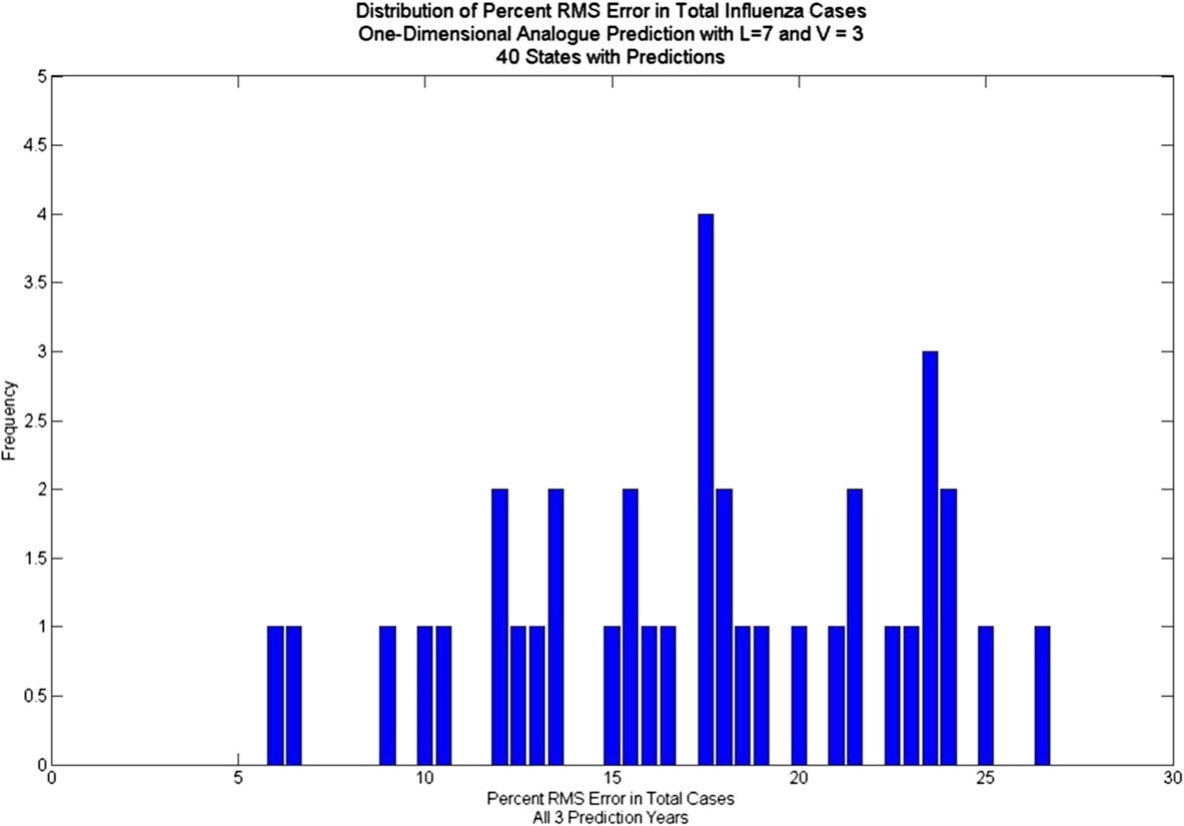
Distribution of percent RMS error in total ILI cases, one-dimensional Analogue prediction

We obtained predictions for all the included states for the 3 years from 2010 to 2013, using the time series up to and including 1 year prior to the prediction date, using ILI incidence only. The predictions consisted of a time series of predicted values of weekly ILI incidence to which we could compare the actual values of ILI incidence. We calculated the metrics and the results appear in Table 1. We also compiled the distribution of RMS errors in both peak height and in total cases. The RMS distribution for peak height appears in Fig. 2 and for total cases appears in Fig. 3.

**Fig. 4:**
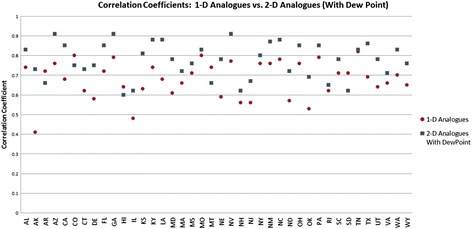
Correlation coefficients for Analogue predictions with dew point vs. 1-dimensional Analogue Prediction

We also calculated the correlation coefficients, in order to compare these methods with previous results using the method of analogues. The correlation coefficients are plotted, along with those for the predictions with dew point, in Fig. 4.

**Fig. 5:**
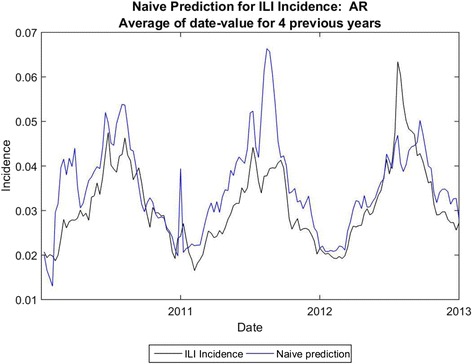
Naïve Prediction for Arkansas

**Fig. 6:**
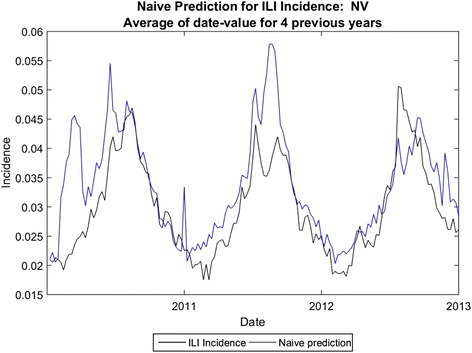
Naïve Prediction for Nevada

**Fig. 7:**
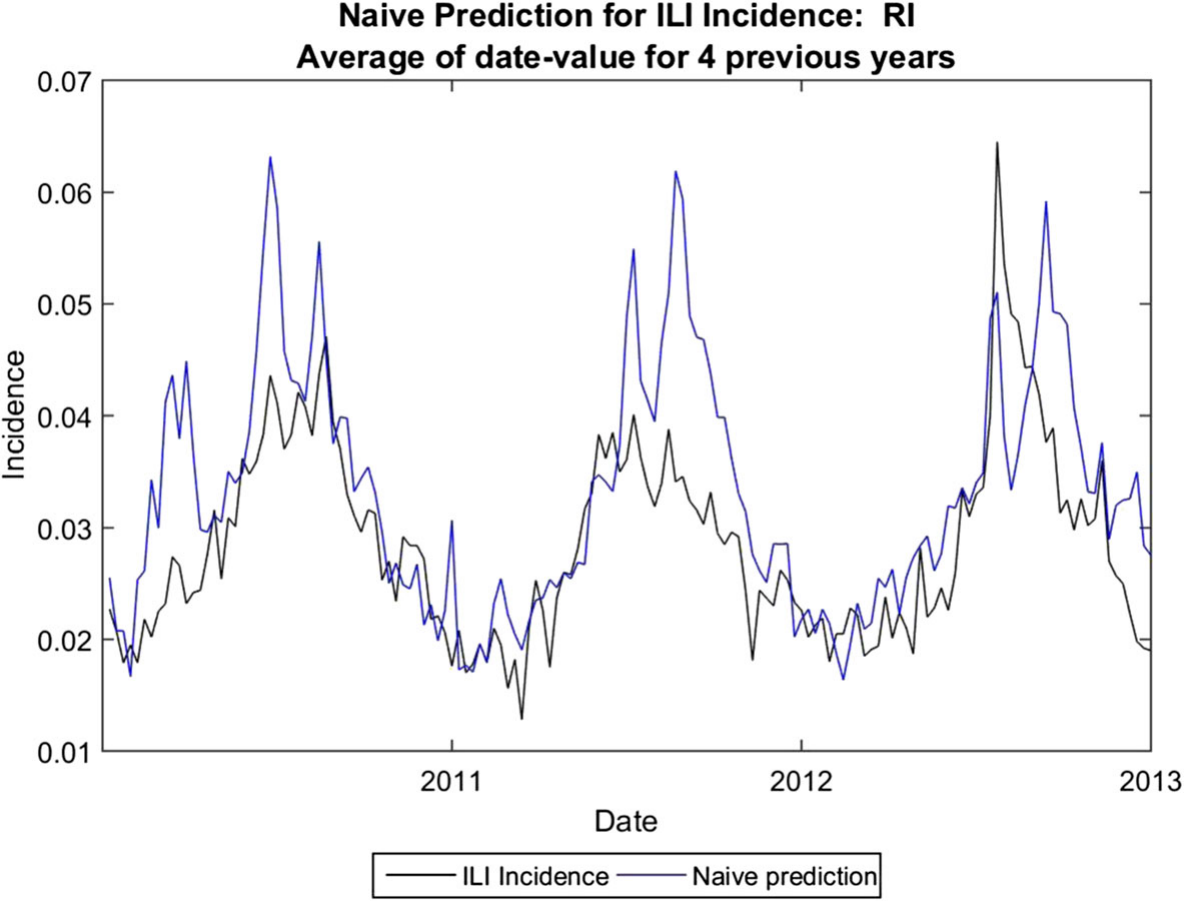
Naïve prediction for Rhode Island

We also used a naïve method of prediction in order to further compare the results from analogue prediction. The naïve method averaged the ILI incidence for the 4 years prior to the prediction date to arrive at the prediction value. Typical prediction sequences are shown in Fig. 5, Fig. 6 and Fig. 7.

The average error for peak height is 14.8 %, and the average error for total cases (area under the curve) is 19.2 % using analogues with parameter optimization. In comparison, the average error for peak height is 25.4 % and the average error for total cases is 13.7 % for the naïve method. Using the parameter optimization the correlation coefficients were comparable, on average, to those obtained by [7] for three-week ahead predictions; the average correlation coefficient was .68 for the four-week ahead predictions, with some states exhibiting higher correlation coefficients (e.g. Colorado) and some with much lower coefficients (e.g. Arkansas). We note that correlation coefficient does not necessarily coincide with error in the prediction of total cases or with error in peak height prediction, however.

## Discussion

**Fig. 8:**
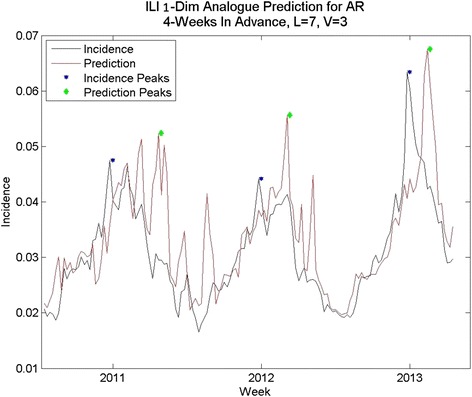
One-dimensional Analogue prediction sequence for Arkansas: “average” RMS error

**Fig. 9:**
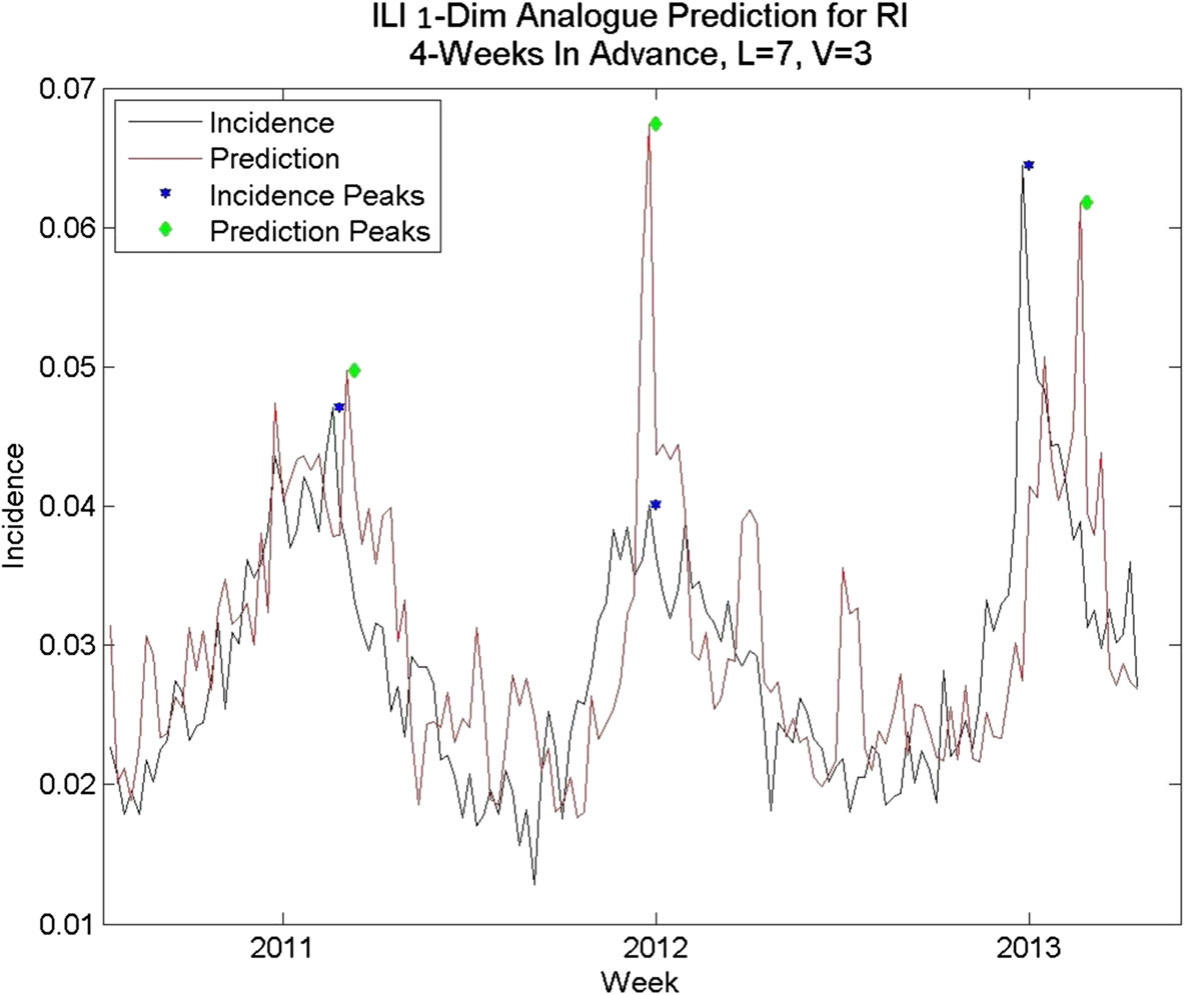
One-dimensional Analogue prediction sequence for Rhode Island: higher-than average percent RMS errors

Comparisons of data vs. prediction for an average prediction (Arkansas) a good prediction (Nevada) and a poor prediction (Rhode Island), based on percent RMS errors, are shown in Fig. 8, Fig. 6 and Fig. 9, respectively. The Arkansas and Nevada prediction curves, in spite of reasonable percent RMS errors, exhibit spurious mid-year spikes that do not coincide with spike in the data. In all three states, the peak of the prediction curves typically does not coincide in time with the real peak, although the percent RMS *height* differences are lower. The Rhode Island curve exhibits many spikes that do not appear in the data. The spread of the distribution of RMS errors (Fig. 2) in height of the yearly peak is further evidence. Although the bulk of the distribution indicates errors of less than 20 % there are some large errors up to 35 %. Regarding the distribution of RMS errors in total cases, there are few states with %RMS error above 20 % and the distribution clusters around a point less than 20 %.

**Fig. 10:**
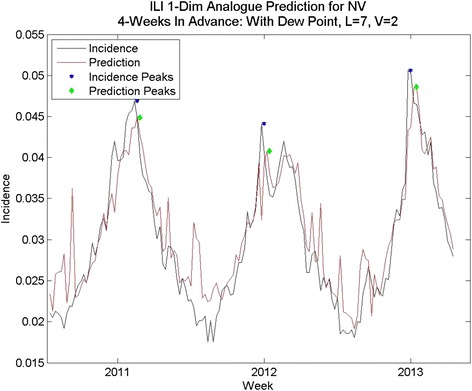
Analogue predictions with dew point, Nevada. The RMS errors for peak height and total cases are slightly higher using dew point, but the correlation coefficients are much higher. Compare with Fig. 11

In some states naïve prediction the percent error for area under the curve was lower than for analogue prediction with dew point. However, the naïve method of prediction was prone to spurious peaks as was the one-dimensional analogue prediction, shown in Fig. 6, vs. the analogue prediction with dew point shown in Fig. 10. Although the percent error for total cases was only slightly lower for the analogue prediction with dew point, the week-to-week prediction is closer to the real values for the analogue prediction with dew point. The running RMS percent error captures this difference better.

**Fig. 11:**
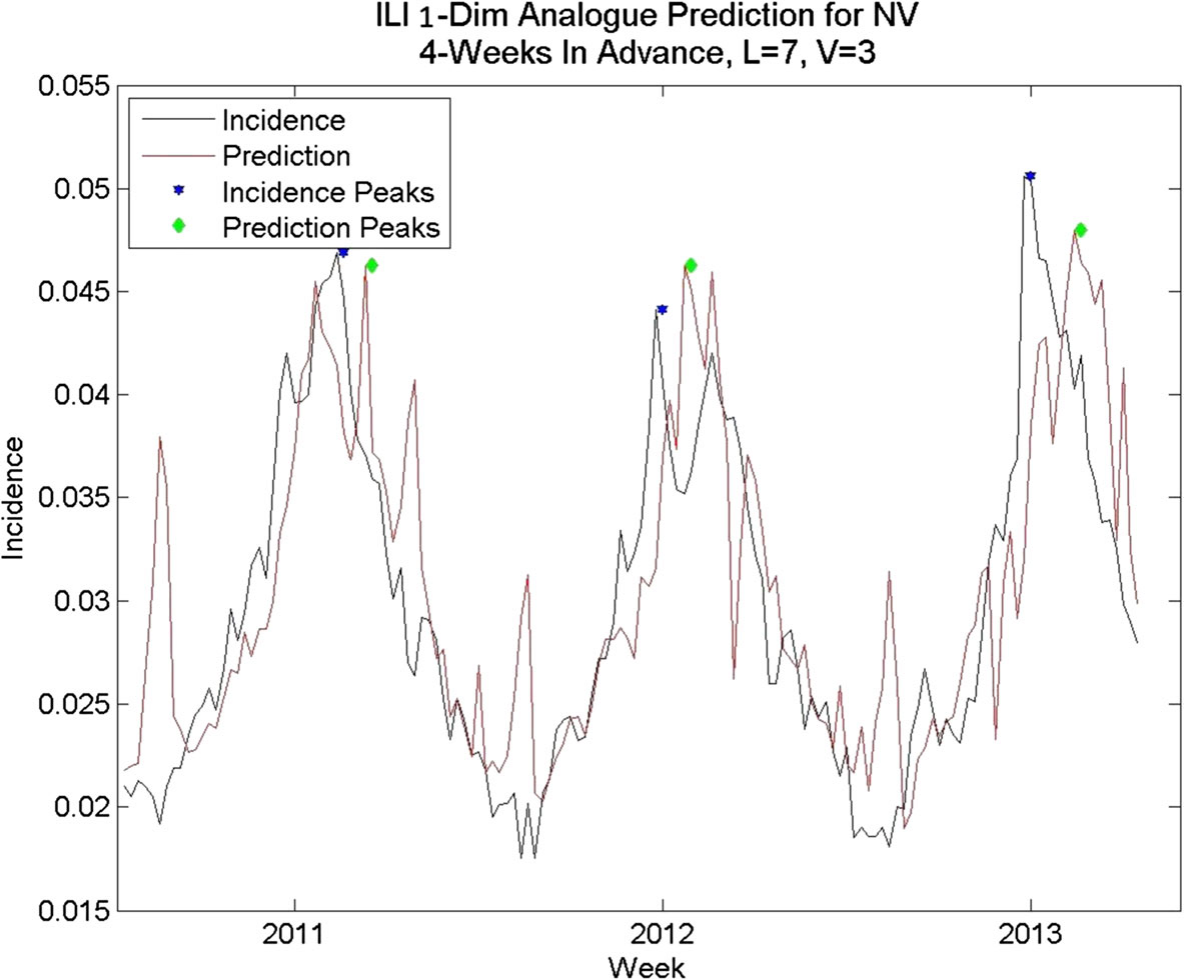
One-dimensional Analogue prediction sequence for Nevada: lower-than-average percent RMS errors

While these predict some states’ peak height and total incidence with less than 20 % error, performance is inconsistent, and often not better than the naïve method. The time series predictions are not close to the time series, the predicted peaks are not close in time to the peaks in the data, and many of the predictions are prone to spurious peaks mid-year. Thus, some improvement is desired. Because the method of analogues’ success depends on finding analogous sequences to time-advance to the prediction point, the only change that is possible to make to this method is to refine the choice of sequences through the addition of other information to better describe the underlying dynamics.

Results from using the dew point to locate dynamically close near-neighbors are significantly better than the results from the one-dimensional analogue predictions. These results appear in Table 2. We note that although nearly all states exhibited good results with parameters *l* = 7 and *v* = 2, there are some states that also performed well (reducing either peak difference or difference in total cases better) with other parameters; those are shown in Table 3.

**Table 2:** Analogue prediction results with dew point

State	*l*	*v*	Peak RMS % Error	AUC RMS % Error	Correlation Coefficient
AL	7	2	11.8	10.7	.83
AK	7	2	5.3	13.0	.73
AR	7	2	14.0	7.3	.66
AZ	7	2	11.0	13.9	.91
CA	7	2	5.1	19.4	.85
CO	7	2	7.1	5.1	.75
CT	7	2	9.4	8.3	.73
DE	7	2	13.7	19.8	.75
FL	7	2	14.7	7.0	.85
GA	7	2	13.0	12.0	.91
HI	7	2	9.1	7.7	.60
IL	7	2	10.5	13.8	.62
KS	7	2	8.9	9.3	.81
KY	7	2	12.3	12.7	.88
LA	7	2	9.5	10.4	.88
MD	7	2	14.6	10.7	.78
MA	7	2	11.9	10.1	.72
MS	7	2	6.8	13.7	.76
MO	7	2	10.2	5.1	.83
MT	7	2	6.5	15.8	.66
NE	7	2	15.1	11.4	.78
NV	7	2	5.3	12.9	.91
NH	7	2	22.1	6.6	.62
NJ	7	2	4.4	19.6	.67
NM	7	2	10.9	12.5	.87
NY	7	2	15.6	8.4	.80
NC	7	2	19.6	18.9	.88
ND	7	2	15.8	7.4	.72
OH	7	2	17.7	12.4	.85
OK	7	2	20.1	10.8	.69
PA	7	2	6.7	12.3	.85
RI	7	2	22.8	12.8	.65
SC	7	2	20.5	15.2	.78
SD	7	2	19.4	32.5	.62
TN	7	2	5.9	8.3	.83
TX	7	2	16.3	7.3	.86
UT	7	2	10.7	2.8	.78
VA	7	2	16.9	21.1	.71
WA	7	2	8.0	21.8	.83
WY	7	2	12.2	7.6	.76
Average for all states with *l* = 7,*v* = 2			12.3	12.2	.77

**Table 3:** Analogue predictions results with dew point using alternate parameter choices for selected locations

State	*l*	*v*	Peak RMS % Error	AUC RMS % Error
AL	7	1	14.7	0.5
AZ	6	1	17.6	10.1
CA	7	5	7.7	17.4
KY	6	1	4.1	15.3
MA	6	1	14.0	1.5
NV	4	1	3.4	6.1
NY	4	1	1.3	8.4

**Fig. 12:**
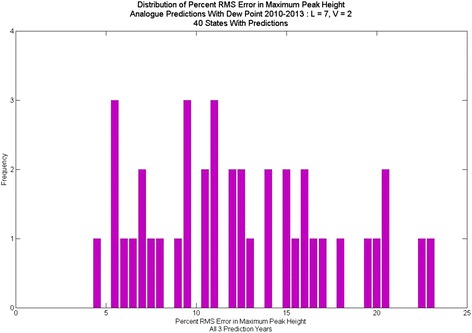
Distribution of percent RMS error in maximum peak height. Analogue prediction with dew point

**Fig. 13:**
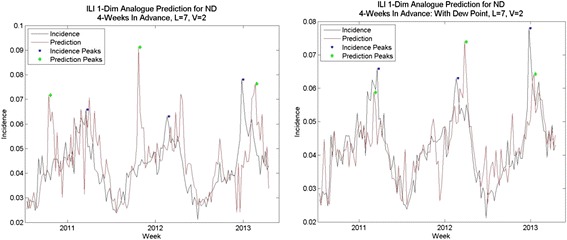
Analogue predictions for North Dakota, with and without dew point. Predictions without dew point had higher-than-average RMS errors; with dew point, average RMS errors. Note that the spurious spike in early 2012 does not occur in predictions with dew point

**Fig. 14:**
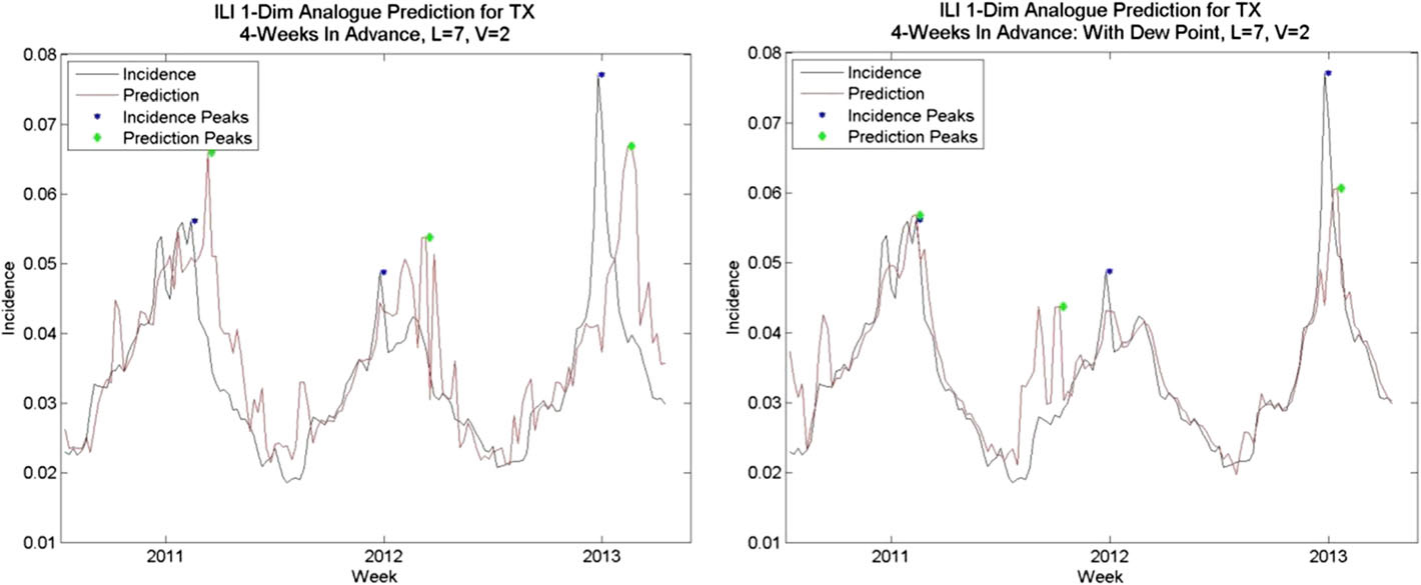
Analogue predictions with and without dew point, Texas. Without dew point, predictions had higher-than-average RMS errors; with dew point predictions had lower-than average RMS errors. Note the predictions for peak are much closer in 2011 and 2013 using dew point

**Fig. 15:**
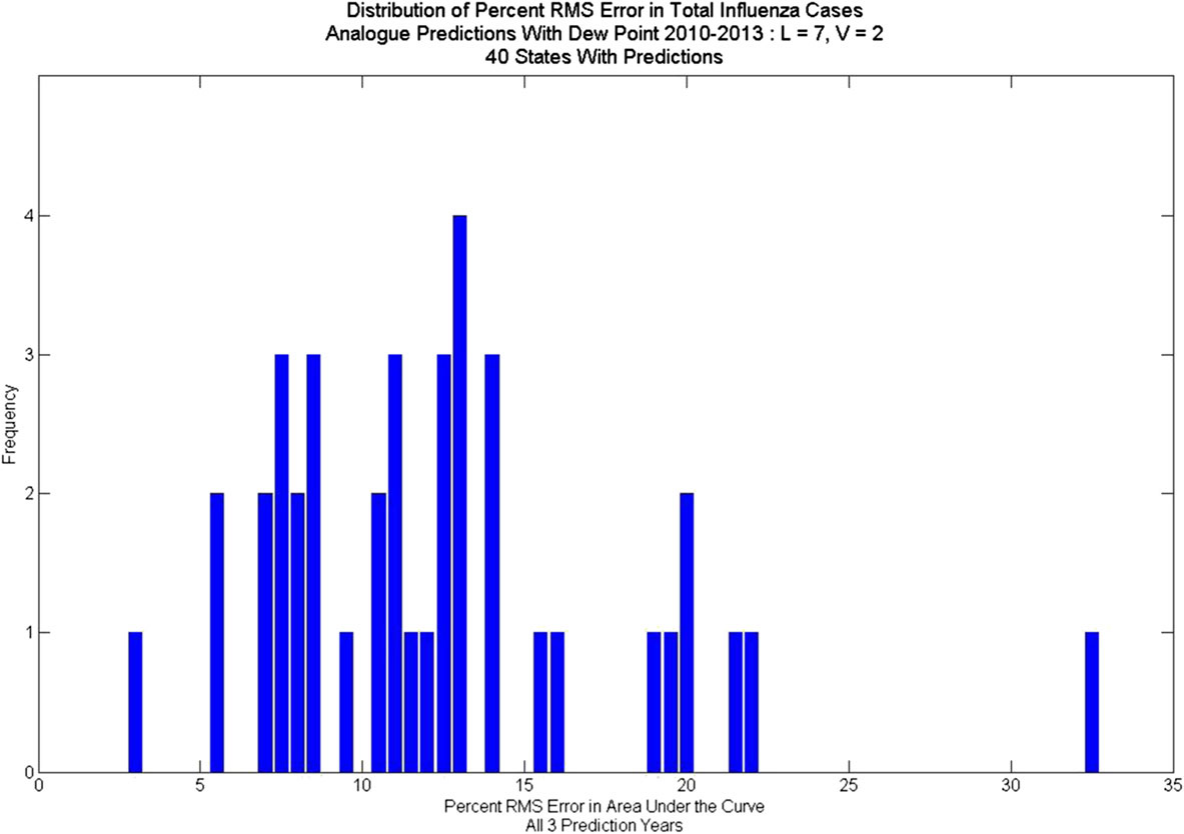
Distribution of percent RMS error in total cases: Analogue prediction with dew point

Contrasting the percent RMS error in peak height for predictions with dew point (Fig. 12, Fig. 13, Fig. 14) to those without (Fig. 2) shows significant improvement with the dew point. Although there is a wide range in the distribution of RMS errors in peak height using dew point, the distributions are centered near 10 % RMS error. Similarly, the distribution of RMS error (Fig. 15) for total cases shows significant spread, but the distribution centers around 12 % (vs. 20 % for one-dimensional prediction without dew point) RMS error.

The table of running error in the area under the curve (eq. 6) is shown in Table 4. This gives an idea of the error that can be expected as ILI cases are predicted prospectively. We see that all but 3 of the states had errors under 10 % for the Analogue prediction with dew point. Errors for the 1-dimensional analogue prediction were variable, with some under 10 % but many above 15 %. Errors for the naïve prediction method were often much larger, in some states above 20 %. Put another way, as predictions are computed each week in a season for the total number of cases so far in the season, the analogue predictions with dew point can be expected to average errors under 10 %. This is not true for the other methods.

**Table 4:** Predictions for average running percent error for total cases (Eq. 6)

State	Naive	1-Dimensional Analogues: (*l = 7,v = 3*)	2-dimensional Analogues (*l = 7,v = 2*)
Alabama	15.9	7.4	6.5
Alaska	30.8	14.7	2.2
Arkansas	18.8	10.3	10.8
Arizona	15.0	6.4	4.9
California	15.9	7.4	2.6
Colorado	23.9	4.9	8.0
Connecticut	11.8	12.1	4.3
Delaware	8.0	4.5	2.2
Florida	12.1	19.8	3.8
Georgia	30.0	9.8	2.5
Hawaii	18.0	12.5	6.4
Illinois	48.6	6.3	3.7
Kansas	21.2	7.0	5.5
Kentucky	15.9	6.5	3.9
Louisiana	9.0	5.1	2.4
Maryland	21.9	14.4	3.8
Massachusetts	5.8	1.5	4.6
Mississippi	15.0	15.8	4.2
Missouri	25.5	7.6	3.9
Montana	18.9	8.7	11.4
Nebraska	17.6	4.1	4.7
Nevada	19.9	5.7	3.6
New Hampshire	6.3	5.9	4.4
New Jersey	6.6	4.1	7.4
New Mexico	21.9	17.4	7.2
New York	9.5	5.8	3.2
North Carolina	14.5	4.8	2.5
North Dakota	13.4	7.8	2.1
Ohio	11.7	6.7	3.8
Oklahoma	17.4	7.9	6.3
Pennsylvania	8.9	3.9	5.7
Rhode Island	19.9	12.9	4.8
South Carolina	38.3	4.6	3.7
South Dakota	14.0	6.4	5.4
Tennessee	6.6	3.3	2.2
Texas	20.4	14.8	6.4
Utah	15.7	12.4	9.7
Virginia	13.3	45.4	11.5
Washington	16.4	14.3	5.2
Wyoming	21.7	18.6	9.9

There are some states for which including the dew point did not improve the correlation coefficient of the predictions, but did decrease the RMS errors on peak height and in total cases (area under the curve). These states include Arkansas and Rhode Island.

Given that some states are quite large and the weather attributes can vary widely from one part of the state to another (for example, Texas includes a desert-like dry area in the west, with a moist subtropical area on the Gulf of Mexico), the data used were an over-simplification of the representative dew point, but were the most accurate data we could obtain at the chosen interval. Thus, we expect that more local dew point forecasts may improve the prediction ability for states with widely varying weather patterns.

This study’s time series included data from 2009, the occurrence of the H1N1 (“swine flu”) pandemic [19]. The rationale behind including these data was that pandemic influenza is as relevant to influenza dynamics as a “typical” influenza season. If a pandemic is to be forecast in the future using this method, sufficient historical sequences need to be present in the data to match future sequences that could be early stages of a pandemic. Those sequences of observations which are not sufficiently close to a current sequence *v* will not be selected as analogues. As long as all available data are used for real-time predictions (and these data include seasons that are not termed “pandemic”), the inclusion of data from “pandemic” years will not affect the accuracy of any predictions that do not give early indications of a pandemic.

Other components that may be relevant to the spread of influenza (and thus the incidence) can also be added to the multidimensional analogues model. For example, virological data may yield increased accuracy of the prediction. These data (influenza type and subtype) are currently tracked by the CDC and are available with the same time lag as incidence, but it is important that they have the same geographic granularity as the incidence and climate data. A national reporting of the matching of the dominant strain to the vaccine may not yield additional accuracy to the analogue predictions because the dynamics of transmission may vary based on the *locally* dominant strain. Thus it is important that these data have the same geographical granularity as the incidence and climate data.

A dynamical model depends heavily on the accuracy and data rate of the data used for it. Currently, weekly incidence is available in most cases [4]. A higher data rate that is commensurate with the typical incubation period of influenza would improve the forecast if available. However, given that the exact time of manifestation of the disease and the ability to report it to a provider contains a lag, a higher data rate may not be more accurate in representing transmission rates. The inclusion of more localized data, however, may give a more accurate representation of the transmission dynamics, particularly if climate data are available with the same temporal and spatial granularity.

## Conclusions

The revised Method of Analogues yielded encouraging results. On paper, these could be sufficient to predict, four weeks ahead, the number of resources (e.g. beds, staff, pharmacy, etc.) that could be required to respond to patient needs in the next four week interval, but results are inconsistent. Although these predictions give relatively low percent RMS error, the inconsistent and spurious spikes would not allow them to be particularly useful as a running prediction of the future during the course of an influenza season. The spread of the distribution of RMS errors in total cases is slightly more encouraging, but there is room for improvement.

Including the dew point as an additional datum with which to choose the analogue sequence for the predictions greatly improved the prediction accuracy in terms of RMS error and correlation coefficient for both the height of the peak and for the total cases. The addition of dew point in general made good predictions better (either in correlation coefficient or RMS errors or both) and made bad predictions much better.

This study shows that the method of analogues can be useful for accurate predictions of the height of influenza season peaks and of the total incidence for the season when climate data are used to refine the prediction sequence. Logical extensions of these results would be both a real-time test of the method with current data and the inclusion of other variables (e.g. observed strains) that may impact the spread of influenza in the subpopulations.

## Abbreviations

AFHSC: Armed Forces Health Surveillance Center
CDC: Centers for Disease Control
DoD: Department of Defense
ILI: Influenza-like illness
RMS: Root mean squared
